# Low Anaerobic Threshold and Increased Skeletal Muscle Lactate Production in Subjects with Huntington's Disease

**DOI:** 10.1002/mds.23258

**Published:** 2010-10-07

**Authors:** Andrea Ciammola, Jenny Sassone, Monica Sciacco, Niccolò E Mencacci, Michela Ripolone, Caterina Bizzi, Clarissa Colciago, Maurizio Moggio, Gianfranco Parati, Vincenzo Silani, Gabriella Malfatto

**Affiliations:** 1 Department of Neurology and Laboratory of Neuroscience, Centro “Dino Ferrari” Università degli Studi di Milano - IRCCS Istituto Auxologico ItalianoMilan, Italy; 2 Department of Neurological Sciences, Centro “Dino Ferrari,” Università di Milano, IRCCS Fondazione Ospedale Maggiore PoliclinicoMangiagalli and Regina Elena, Milan, Italy; 3 Department of Cardiology, S. Luca Hospital, IRCCS Istituto Auxologico ItalianoMilan, Italy; 4 Department of Clinical Medicine and Prevention, Università di Milano-BicoccaMilan, Italy

**Keywords:** Huntington's disease, skeletal muscle, anaerobic threshold, mitochondria

## Abstract

Mitochondrial defects that affect cellular energy metabolism have long been implicated in the etiology of Huntington's disease (HD). Indeed, several studies have found defects in the mitochondrial functions of the central nervous system and peripheral tissues of HD patients. In this study, we investigated the in vivo oxidative metabolism of exercising muscle in HD patients. Ventilatory and cardiometabolic parameters and plasma lactate concentrations were monitored during incremental cardiopulmonary exercise in twenty-five HD subjects and twenty-five healthy subjects. The total exercise capacity was normal in HD subjects but notably the HD patients and presymptomatic mutation carriers had a lower anaerobic threshold than the control subjects. The low anaerobic threshold of HD patients was associated with an increase in the concentration of plasma lactate. We also analyzed in vitro muscular cell cultures and found that HD cells produce more lactate than the cells of healthy subjects. Finally, we analyzed skeletal muscle samples by electron microscopy and we observed striking mitochondrial structural abnormalities in two out of seven HD subjects. Our findings confirm mitochondrial abnormalities in HD patients' skeletal muscle and suggest that the mitochondrial dysfunction is reflected functionally in a low anaerobic threshold and an increased lactate synthesis during intense physical exercise. © 2010 Movement Disorder Society

Huntington's disease is an autosomal-dominant neurodegenerative disorder characterized by chorea, dementia, and psychiatric disturbances. The genetic mutation underlying the disease is the expansion of the triplet cytosine-adenosine-guanosine (CAG) in the *IT-15* gene; this mutation encodes for an expanded polyglutamine (polyQ) tract in the huntingtin (htt) protein.^1^ Htt is ubiquitously expressed in the brain as well as in many extra-CNS tissues such as skeletal muscle.^2^ The expression of mutated htt has deleterious effects on skeletal muscle; in particular, HD patients suffer from muscular weakness^3^,^4^ and undergo progressive muscular wasting.^5^,^6^ In addition to this clinical evidence, various abnormalities have been observed in the muscular tissues of HD patients and in HD mouse models. These abnormalities include skeletal muscle atrophy^7^,^8^ and impairment of adenosine triphosphate (ATP) metabolism, which manifests as a reduced ratio of phosphocreatine to inorganic phosphate and a lower production of mitochondrial ATP.^9^–^11^ Whereas mitochondria-related energetic dysfunctions have been found in both the CNS and skeletal muscle of HD patients,^12^ it is still unclear whether the cellular energy metabolism impairment is a primary event in the cascade of pathogenic events that occurs in the brains of HD patients. To clarify this issue, previous studies used magnetic resonance spectroscopy (MRS) to analyze the brain lactate levels of symptomatic and presymptomatic HD subjects; these studies, however, produced conflicting data.^13^–^16^ As skeletal muscle cells, like neurons, are postmitotic cells that are highly dependent on oxidative metabolism, we decided to investigate the in vivo oxidative metabolism of exercising muscle in HD subjects. We hypothesized that during physical exercise, the lower level of ATP synthesis in HD patients would reduce the ability of muscle cells to extract O_2_ from blood; as a result, HD patients would reach the anaerobic threshold (AT) early and show a correspondingly high level of lactate production.^17^,^18^ This clinical study is designed to measure ventilatory and cardiometabolic parameters as well as lactate production in presymptomatic and symptomatic HD gene carriers during a cardiopulmonary exercise test.

## PATIENTS AND METHODS

### HD Patients, Presymptomatic HD Subjects, and Control Subjects

Table 1 shows the clinical and demographic data of the HD patients and control subjects. All of the HD subjects had had DNA analysis demonstrating more than 39 CAG repeats. Patients were excluded if they met any of the following criteria: (1) concomitant presence of metabolic, endocrine or muscular disorders; (2) arterial hypertension that required treatment (defined as a systolic pressure >140 mm Hg and a diastolic pressure >90 mm Hg); (3) systolic and/or diastolic heart failure (defined as an ejection fraction <50% and/or an abnormal diastolic phase) and valvular or morphological abnormalities diagnosed through echocardiography; (4) use of drugs that affect metabolism and/or muscular functions;(5) a history of drug addiction (6) a body mass index (BMI) <18 or >25 and (7) an inability to use the bicycle ergometer. All HD patients were able to walk without assistance and able to independently carry out activities of daily living.

**TABLE 1:** Demographic, clinical, and genetic data of HD patients (nine males and six females), presymptomatic subjects (seven males and three females), and healthy controls (16 males and nine females)

	Symptomatic HD patients (N = 15)	Presymptomatic HD subjects (N = 10)	Controls (N = 25)
Age (yr)	48.2 ± 10.2 (29–67)	37.6 ± 6.7 (21–45)	43.7 ± 10.6 (31–70)
CAG triplet number	45.3 ± 3.2 (41–52)	43.8 ± 2.5 (42–49)	–
Age at onset (yr)	44.7 ± 10.9 (28–65)	–	–
Duration of illness (yr)	3.9 ± 3.1 (1–10)	–	–
UHDRS part I	31.0 ± 12.2 (17–53)	–	–
Total functional capacity	10.7 ± 2.2 (6–13)	–	–

Data are expressed as mean ± SD (range).

Controls were selected from healthy volunteer subjects according to the same exclusion criteria. Mean age of the control group did not significantly differ from mean age of presymptomatic and symptomatic HD group. Mean age of presymptomatic group was lower as compared to symptomatic HD group (*P* < 0.05). Presymptomatic-HD and control groups both included subjects that had a sedentary life style and subjects that performed moderate physical activity (1–2 hours physical exercise/week).

Each subject gave his or her written consent after being fully informed of any risks and discomfort associated with participation in the study. The study was approved by the Ethics Committee of the Istituto Auxologico Italiano, and all study procedures followed the recommendations of the Helsinki Declaration of 1975.

### Exercise Protocol

All subjects rested for 45 minutes before beginning the exercise portion of the study. The exercise test was performed on an electrically-braked bicycle according to a validated protocol.^19^ A cardiopulmonary exercise system (Sensor Medics V2900, USA) was used to monitor breath-by-breath measurements of VE (expired ventilation), VO_2_ and VCO_2_. Derived entities such as VE for O_2_ and CO_2_ (VE/VO_2_, VE/VCO_2_), the respiratory quotient VCO_2_/VO_2_ and respiratory rate per minute were presented on-line. The equipment was calibrated before every test. A 12-lead ECG was used to monitor for arrhythmia and ST segment changes. The test began with 2 minutes of variable sampling whereas the subject was at rest and was followed by 2 minutes of unrestricted exercise. The workload was increased by 25 Watts every 2 minutes. The exercise test was symptom-limited and used a Borg scale (from 0 to 10) to rate dyspnoea, fatigue and chest pain. The subjects were encouraged to exercise until they were exhausted. Blood pressure and heart rate were measured every 2 minutes. All respiratory parameters were measured from plots over time, resulting in moving average values. The peak VO_2_, VE/VO_2_, and VE/VCO_2_ were the last of three values that were recorded during the final 30 seconds of exercise. If this last value was not the highest, the mean of the last three values was calculated. The anaerobic threshold was calculated according to the V-slope method. After the test, patients rested in a supine position for at least 5 minutes. The following exercise parameters were evaluated in all subjects:

1. Exercise/cardiac parameters:
  - Maximal ergometric working capacity (Wpeak), defined as the maximal work (Watts) reached for at least 1 minute
  - Peak exercise heart rate (HRpeak) and heart rate at the anaerobic threshold (HR AT)
  - Peak VO_2_/kg (mL/Kg/min), i.e., the maximal oxygen consumption, expressed both in absolute values (ml/Kg/min) and as a percent of theoretical maximum capacity according to age, body type, and sex (peak VO_2_ %)
  - O_2_ pulse (ml/beat) both at anaerobic threshold (AT pO_2_) and at the exercise peak (peak pO_2_)
  - Aerobic threshold (AT VO_2_), expressed as an absolute value (ml/Kg/min), as a percent of the predicted maximum (AT%) and as Watts reached (AT Watts)
2. Ventilatory variables:
  - Respiratory quotient at the anaerobic threshold (RQ AT) and at peak exercise (RQ peak)
  - Ratio of dead space to tidal volume (VD/Vt)
  - Ratio of ventilation to CO_2_ production at peak exercise (peak VE/VCO_2_)

### Blood Sampling and Lactate Concentration Assay

A peripheral antecubital venous access was positioned before the beginning of the test. Blood samples were drawn whereas the subject was at rest and at the beginning of each 2-minute incremental step during the exercise. The lactate concentration of the plasma was assessed using a colorimetric assay (Lactate Reagent, Trinity Biotech, Ireland).

### Muscle Biopsies

Informed consent was obtained from each patient. Open muscle biopsies were obtained at rest from the biceps brachii muscle of patients through a small surgical incision under local anesthesia.

### Human Muscular Cultures

Human myoblast cultures were obtained from biopsy specimens (supporting information Table 1) as previously described.^20^ Equal numbers of myoblasts were plated on 100-mm dishes in 10 ml of culture medium. The media and cells were collected 24 hours later. The media were assayed for lactate concentration and the cells were counted using Coulter Counter cell (Beckman, CA).

### Morphological Studies

We examined skeletal muscle biopsies from seven HD patients. For light microscopy studies, cryostat cross sections were processed according to standard histological (Gomori's Trichrome, H&E) and histochemical (COX, SDH, double staining for COX, and SDH) techniques.^21^ A small part of each bioptic sample was fixed in 2,5% glutaraldehyde (pH 7,4), postfixed in 2% osmium tetroxide and then embedded in Spurr's resin for ultrastructural examination. Finally, ultrathin sections were stained with lead citrate and uranyl acetate and examinated with Zeiss EM109 transmission electron microscope.^21^

### Statistical Analysis

A Kolmogorov-Smirnov test was used to test the data for normality and a Levene test was used to verify the homogeneity of group variances. Cardiopulmonary parameters and blood lactate concentrations were compared with an analysis of variance (ANOVA) procedure using a Tukey test. Pearson or Spearman correlation coefficients were used to test for correlations between clinical and genetic data and the cardiopulmonary parameters. Lactate concentrations in myoblast culture media were compared with a Kruskal-Wallis ANOVA followed by Dunn's test.

## RESULTS

### Cardiorespiratory Measurements

All of the subjects completed the exercise test without complications. In all subjects, the peak RQ was close to 1, showing a truly maximal test. No arrhythmias or ST changes suggestive of ischemic problems were detected in HD or control subjects during the exercise. The cardiopulmonary test parameters of HD patients, presymptomatic HD subjects and healthy controls are reported in Table 2.

**TABLE 2:** Cardiopulmonary test parameters of HD patients, presymptomatic HD subjects, and healthy controls

	Symptomatic HD patients	Presymptomatic HD subjects	Controls	
*W*_peak_ (Watts)	111.7 ± 37.6; (75–200)	165.0 ± 39.4; (125–225)	158.7 ± 45.8; (100–250)	*P* = 0.003 s-HD vs C
Peak VO_2_/kg (mL/Kg/min)	23.4 ± 6.7; (14.4–39.1)	29.5 ± 7.0; (19.6–42.2)	28.8 ± 6.0; (19.7–47.5)	*P* = 0.026 s-HD vs C
Peak VO_2_/kg (% of theorethical)	75.7 ± 22.3; (42–125)	78.7 ± 21.1; (53–113)	83.3 ± 14.7; (60–129)	
Heart rate peak (beats/min)	145.6 ± 19.4; (106–180)	156.3 ± 11.0; (139–176)	154.3 ± 18.9; (112–185)	
RQ (peak) adimensional ratio	1.0 ± 0.1; (1.0–1.1)	1.1 ± 0.1; (0.9–1.3)	1.1 ± 0.2; (0.9–1.7)	
O_2_ pulse peak (mL/beat)	11.3 ± 4.2; (5.8–20.4)	13.5 ± 3.7; (8.2–18.3)	13.4 ± 3.5; (6.9–18.8)	
O_2_ pulse peak (%)	84.5 ± 19.7; (49–128)	93.1 ± 19.0; (73–135)	100.0 ± 21.6; (62–140)	
VD/VT (%)	97.3 ± 36.5; (57–187)	72.6 ± 17.6; (40–92)	66.5 ± 19.4; (30–104)	
VE/VCO_2_ adimensional ratio	32.0 ± 2.9; (27–36)	30.1 ± 4.3; (24–38)	29.9 ± 3.2; (26–36)	
AT VO_2_ (mL/Kg/min)	13.3 ± 2.5; (9.7–19.0)	13.6 ± 3.3; (9.8–20.3)	19.0 ± 5.0; (11.9–33.1)	*P* = 0.000125 s-HD vs C
				*P* = 0.000125 preHD vs C
AT (%)	38.9 ± 7.3; (27–50)	35.3 ± 8.0; (26–56)	54.7 ± 13.1; (39–88)	*P* = 0.000251 s-HD vs C
				*P* = 0.000166 preHD vs C
AT (Watts)	38.3 ± 12.9; (25–50)	57.5 ± 16.9; (25–75)	99.0 ± 43.0; (50–200)	*P* = 0.000002 s-HD vs C
				*P* = 0.000002 preHD vs C

Data are expressed as mean ± SD; (range). Not reported statistical scores were *P* > 0.05.

**FIG. 1:**
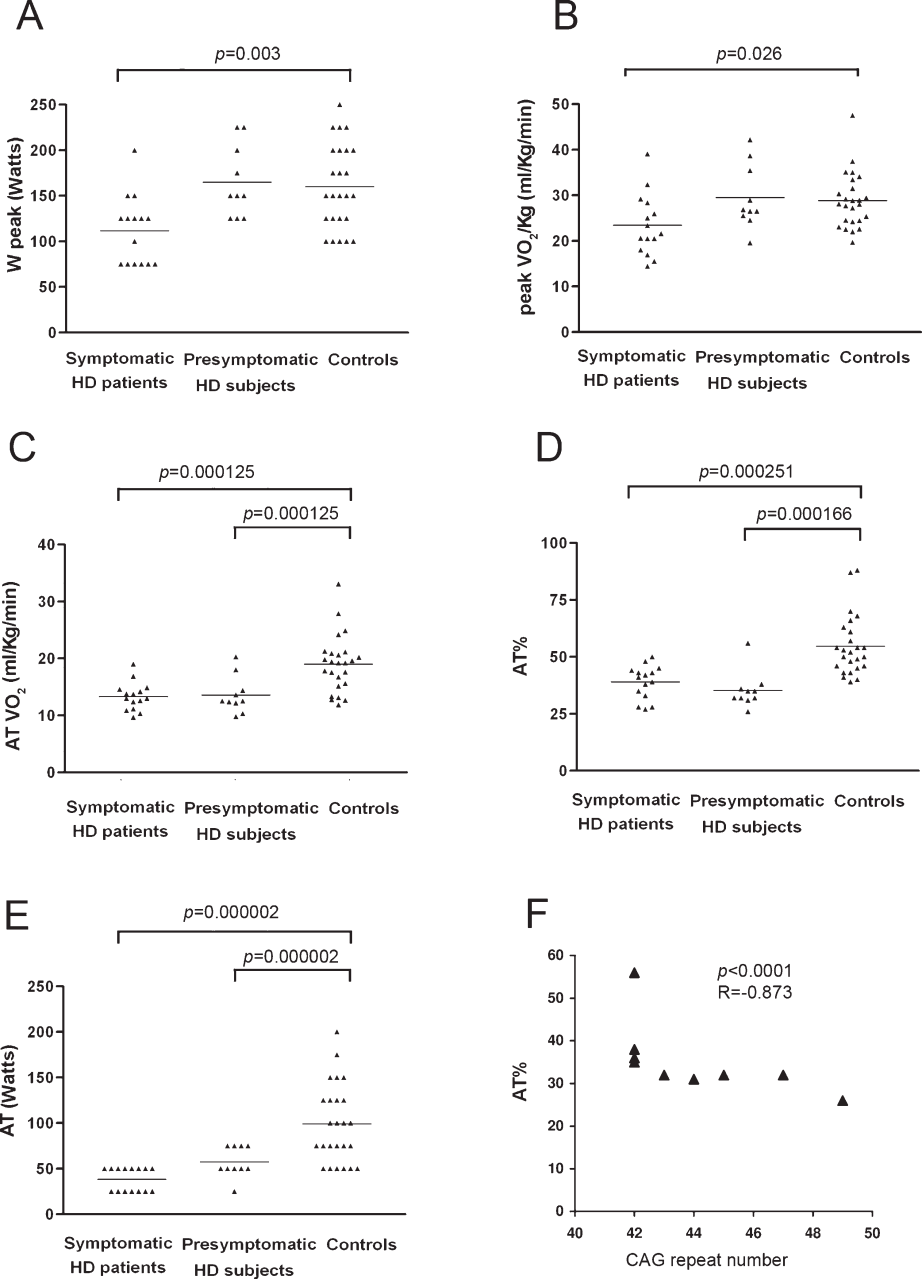
**(A)** Scatter plot of maximal ergometric working capacity values (Wpeak) and **(B)** maximal oxygen consumption, expressed as absolute values (Peak VO_2_) in HD patients (N = 15), presymptomatic subjects (N = 10) and control subjects (N = 25). **(C)** Scatter plot of aerobic threshold values expressed as absolute value (ATVO_2_), **(D)** as percent of the predicted maximum (AT%) and **(E)** as Watts reached (AT Watts). Mean values are indicated with horizontal bars. (F) Scatter plot graph showing that AT% correlates with CAG repeat number in presymptomatic HD subjects. The graph shows nine dots because two subjects had identical AT% and CAG repeat number.

The peak power (Wpeak) and peak oxygen consumption (peak VO_2_) values were significantly reduced in symptomatic HD patients as compared to controls (Table 2, Fig. 1A,B). No difference in maximal exercise capacity was detected between presymptomatic HD and control subjects. Notably, there was no difference in O_2_ peak pulse, VD/Vt or VE/VCO_2_ among the groups; this data indicates a normal cardiac and ventilatory performance in all the groups.^22^

The anaerobic threshold values were significantly lower in the symptomatic and presymptomatic HD subjects than in the control subjects; this was true for all measurements, including the absolute value (ATVO_2_, mL/Kg/min), percent of the predicted maximum (AT%) and Watts reached (AT, Watt) (Table 2, Fig. 1C–E). We examined the data for a potential correlation between the cardiopulmonary test parameters and clinical or genetic data of the HD subjects. In the symptomatic HD patients, no significant correlation was found among clinical data (age at onset, duration of illness, UHDRS I, and TFC), genetic data (CAG repeat number), and Wpeak, ATVO_2_, AT%, or AT. Notably, a significant negative correlation was found between AT% and CAG repeat number in presymptomatic HD subjects (Fig. 1F; *P* < 0.0001; *R* = −0.873, Spearman Correlation).

### Blood Lactate Accumulation During a Cycloergometric Test and Lactate Production from In vitro Muscular Cell Cultures

**FIG. 2:**
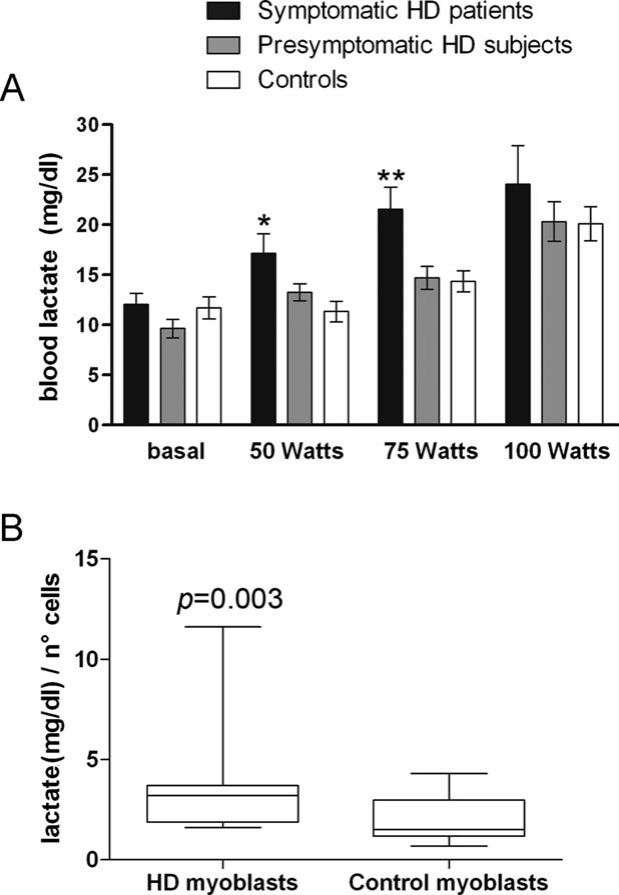
(A) Lactate concentrations in blood (mean ± SD) during cardiopulmonary test (*P = 0.021; **P = 0.014 vs. controls). (B) Graph representing the median and the percentiles of lactate concentrations in cell culture media. Data were expressed as mg/dL and normalized on cell number. The ends of the boxes define the 25th and 75th percentiles, with a line at the median and error bars defining the 10th and 90th percentiles. Medians were: HD cells 3.0 mg/dL/number of cells. Control cells: 1.6 mg/dL/number of cells (*P* = 0.003 vs. control cells).

Figure 2A shows mean values of blood lactate concentrations at the various levels of work. The plasma lactate values did not differ between the symptomatic HD patients and the control subjects when they were at rest; however, at 50 and 75 Watts the mean plasma lactate concentration was significantly higher in symptomatic HD patients than in the controls (50 Watts, *P* = 0.021 vs controls; 75 Watts, *P* = 0.014 vs controls). Presymptomatic HD subjects had a mean lactate value at 50 Watts that was higher than that of the controls, but the difference was not statistically significant.

To determine whether the increased lactate production was related to a primary defect in the mitochondrial function of muscular cells, we measured lactate production in *in vitro* muscular cell cultures from five HD patients and five age-matched controls (the biopsy data are reported in supplemental Table 1). The lactate concentration in the media of the HD cultures was significantly higher than in the media of the control cultures (Fig. 2B).

### Histochemistry and Ultrastructural Studies of HD Skeletal Muscle

**FIG. 3:**
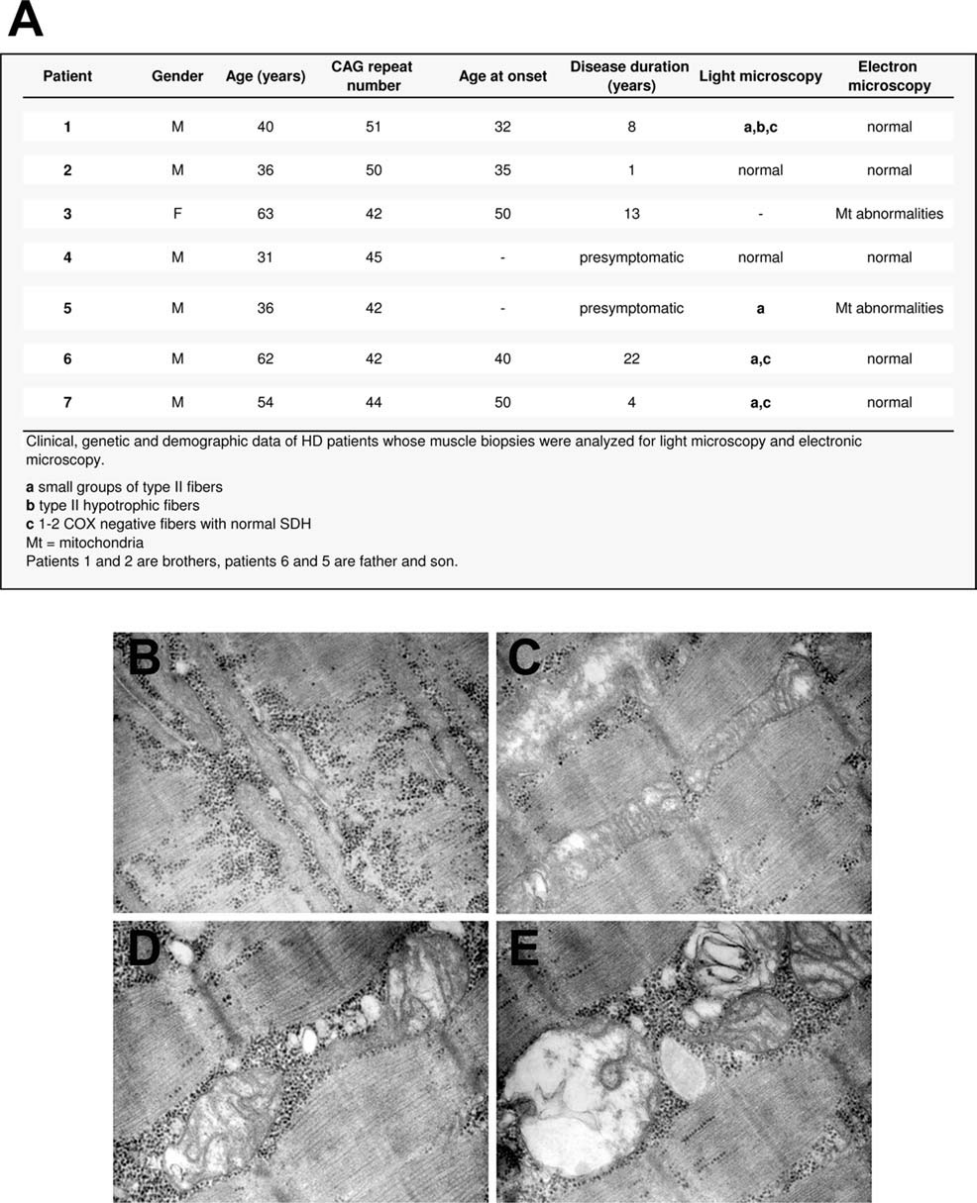
**(A)** Data of muscle biopsies analyzed for light microscopy and electronic microscopy. **(B-E)** Electron microscopy results (Part 3) (20000 X). Abnormally elongated mitochondria with derangement of cristae and vacuoles (B and C). Swollen mitochondria with progressive loss of matrix substance (D and E) and disruption of residual cristae (E).

We examined six out of seven muscle biopsies (sample n 3 was too small for reliable examination, Fig. 3A) and we found small groups of type II fibers in patients 1, 5, 6, and 7, patient n 1 also presenting scattered type II hypotrophic fibers. We detected no significant oxidative alterations except for the presence of 1–2 COX-negative fibers, without mitochondrial proliferation (normal SDH), in patients n 1, 6, and 7.

In two patients (n 3 and 5), ultrastructural studies showed a consistent number of abnormally elongated mitochondria with derangement of cristae and vacuoles (Fig. 3B,C). Also, some mitochondria gradually become swollen with progressive loss of matrix substance and disruption of residual cristae (Fig. 3D,E).

## DISCUSSION

Our study shows that symptomatic HD subjects have a reduced work capacity (Wpeak) during a cardiopulmonary test. This data complements the recent reported of a significant reduction in muscle strength in symptomatic HD patients.^4^ Presymptomatic HD subjects had normal Wpeak values during the same exercise test, which suggests that the Wpeak reduction in symptomatic patients may be related to the reduction in muscle bulk that occurs as the disease progresses.^5^,^6^ Our findings confirm a strength deficit in HD patients and support the idea that physical therapy aimed at improving muscle strength could benefit these patients, particularly during the early stages of the disease.^23^

This study shows that low anaerobic threshold (AT) values and an early increase of blood lactate are linked to HD. Both symptomatic and presymptomatic HD subjects had an anticipated AT during the incremental exercise. The AT is an index normally used to estimate exercise capacity. During the initial (aerobic) phase of cardiopulmonary exercise, expired ventilation (VE) increases linearly with VO_2_ and reflects aerobically produced CO_2_ in the muscles. During the latter phase of exercise, anaerobic metabolism occurs when the oxygen supply cannot keep up with the increasing metabolic requirements of exercising muscles. At this time, there is a significant increase in lactic acid production in the muscles and in the blood lactate concentration.^24^

In our opinion, the low AT values and elevation of blood lactate in HD subjects reflect abnormalities in O_2_ utilization; this is consistent with abnormal oxidative metabolism in skeletal muscle. Presymptomatic subjects did not show a reduction in Wpeak values, which suggests that lower AT% values are not correlated with muscular atrophy. Notably, our data highlighted an inverse correlation between AT% values and CAG repeats in HD gene carriers; this data strongly suggests that mutant htt directly results in deficits in the mitochondrial respiratory chain, even in presymptomatic HD patients. Among symptomatic HD patients, the CAG repeat number was not significantly correlated with AT% values. This data indicates that factors other than the CAG repeat number (such as muscular atrophy) may also contribute to AT% reduction in the more advanced stages of the disease. Several studies have suggested that the work rate corresponding to the AT could be used as an index for determining the optimal training intensity,^25^ therefore the information gathered in this study suggests that a cardiopulmonary test should be included in the physical therapy program for HD subjects.

Our examination of skeletal muscle tissue from six HD subjects with an histochemical marker for mitochondrial oxidative function (COX) did not reveal any significant abnormalities in both presymptomatic and symptomatic subjects. This data agrees with previously reported histological and histochemical examination of muscle biopsies from HD subjects.^6^ Given the previously reported observations of structural mitochondrial abnormalities in cortical biopsies from HD patients^26^–^28^ and in the muscle biopsy from one HD gene carrier,^3^ we performed electron microscopy examination of HD muscle biopsies. Interestingly, we found abnormally elongated mitochondria with cristae derangement and vacuoles in two specimens (pt n 3 and 5). These findings are similar to those described.^28^,^29^ Pt n 3 is a 63-years-old woman with a disease duration of 13 years and 42 CAG repeats, whereas pt n 5 is a presymptomatic 36-years-old man with 42 repeats. Interestingly, his father (pt n 6), who has the same number of repeats and a disease duration of 22 years, does not show structural mitochondrial changes. We hypothesize that, as reported,^28^ the same mitochondrial alterations could be present at central nervous system level also in patients who do not show skeletal muscle abnormalities. Also, a possible explanation for the finding of mitochondrial alterations in few subjects is that these alterations may correlate with the lifestyle of the patients and may be more evident in physically active subjects than in more sedentary, possibly older, ones.

Conflicting data have been reported about cardiac dysfunction in HD.^12^ Indeed, mutant htt has been blamed for cardiotoxic effects in mouse models, including heart atrophy^7^ and defects in contractile functions.^30^ Nevertheless, epidemiological studies have not found heart disease to be more common in HD patients than in controls.^31^

In this study, the patients' cardiopulmonary response to exercise did not resemble the pattern that is typical of patients with heart failure. At peak exercise, the HD patients showed a normal O_2_ pulse, which suggests a normal cardiac output^22^; in addition, they had a normal ventilatory response, with VE/VCO_2_ values below the cutoff-value of 35.^32^ These results do not show an increased risk for cardiac disease in HD patients. Rather, the response of HD patients to cardiopulmonary testing suggests a primary defect in the muscular energetic metabolism. The increased lactate production we found in HD myoblast cultures further highlights the inadequate mitochondrial oxidative respiration of HD muscle and agrees with our previous reports showing mitochondrial dysfunction in HD myoblasts.^20^,^33^

Finally, we believe that AT measures could be useful as in vivo assays during the screening of drugs designed to improve mitochondrial function in HD patients. For example, a deficit in PGC-1α (peroxisome proliferator-activated receptor-γ coactivator 1α), a transcriptional coactivator implicated in mitochondrial biogenesis, was recently found in both the brain^34^ and skeletal muscles^35^ of HD patients. Molecules that activate PGC-1α may be therapeutically useful,^36^ and in vivo AT measures in HD subjects could help to evaluate a potential drug's benefits.

## Financial Disclosures:

All the Authors do not have financial support to disclose for the past 12 months.

**Author Roles:** Andrea Ciammola made clinical diagnoses and collected clinical data; Jenny Sassone performed genetic diagnoses; Jenny Sassone and Clarissa Colciago prepared the primary mioblasts from patient's biopsies, carried out the experiments with primary mioblasts, and performed lactate dosage in plasma and cell culture medium; Monica Sciacco, Michela Ripolone, and Maurizio Moggio carried out histochemical analyses and electronic microscopy on muscle biopsies; Caterina Bizzi and Gabriella Malfatto performed cardiopulmonary tests and collected ventilatory, and cardiometabolic parameters; Jenny Sassone performed statistical analysis; Vincenzo Silani performed muscular biopsies on HD subjects and reviewed the manuscript; Andrea Ciammola, Vincenzo Silani, and Gianfranco Parati provided financial support; Andrea Ciammola, Jenny Sassone, and Gianfranco Parati conceived and designed the study; Andrea Ciammola, Jenny Sassone, and Gabriella Malfatto wrote the paper; all others received, edited, and approved the manuscript.
