# Supplementary material for: Low Anaerobic Threshold and Increased Skeletal Muscle Lactate Production in Subjects with Huntington's Disease
Source: Mov Disord. 2010 Oct 7;26(1):130–7. doi: 10.1002/mds.23258 (PMC3081141; doi:10.1002/mds.23258)
Supplement: Supplementary file 1 [file mds0026-0130-SD1.pdf]

Subjects no.	Genotype	Gender	Age (years)	CAG repeat number	Age at onset	Independance scale score	Functional capacity
**1**	HD	M	40	55	31	70	4
**2**	HD	M	36	50	35	90	11
**3**	HD	M	55	43	40	70	6
**4**	HD	M	53	47	49	100	13
**5**	HD	M	58	42	50	50	2
**6**	Control	M	36				
**7**	Control	M	50				
**8**	Control	M	48				
**9**	Control	M	27				
**10**	Control	M	48				

Supplemental Table 1. Demographic and genetic data of HD patients and control subjects. Muscle biopsies were used for myoblast culture.
